# The Dynamics of Latifundia Formation

**DOI:** 10.1371/journal.pone.0082863

**Published:** 2013-12-20

**Authors:** Luis Fernando Chaves

**Affiliations:** 1 Graduate School of Environmental Sciences, Hokkaido University, Sapporo, Japan; 2 Programa de Investigación en Enfermedades Tropicales (PIET), Escuela de Medicina Veterinaria, Universidad Nacional, Heredia, Costa Rica; 3 Institute of Tropical Medicine (NEKKEN), Nagasaki University, Nagasaki, Japan; Cinvestav-Merida, Mexico

## Abstract

Land tenure inequity is a major social problem in developing nations worldwide. In societies, where land is a commodity, inequities in land tenure are associated with gaps in income distribution, poverty and biodiversity loss. A common pattern of land tenure inequities through the history of civilization has been the formation of latifundia [Zhuāngyuán in chinese], i.e., a pattern where land ownership is concentrated by a small fraction of the whole population. Here, we use simple Markov chain models to study the dynamics of latifundia formation in a heterogeneous landscape where land can transition between forest, agriculture and recovering land. We systematically study the likelihood of latifundia formation under the assumption of pre-capitalist trade, where trade is based on the average utility of land parcels belonging to each individual landowner during a discrete time step. By restricting land trade to that under recovery, we found the likelihood of latifundia formation to increase with the size of the system, i.e., the amount of land and individuals in the society. We found that an increase of the transition rate for land use changes, i.e., how quickly land use changes, promotes more equitable patterns of land ownership. Disease introduction in the system, which reduced land profitability for infected individual landowners, promoted the formation of latifundia, with an increased likelihood for latifundia formation when there were heterogeneities in the susceptibility to infection. Finally, our model suggests that land ownership reforms need to guarantee an equitative distribution of land among individuals in a society to avoid the formation of latifundia.

## Introduction

The socialized nature of ecosystem transformation is a major theme of study within the broad field of environmental studies [1], [2], [3]. Over recent years the fields of ecology and epidemiology have become aware of the fundamental role humans play on ecosystem transformation [3] and the impacts of such transformations on biodiversity loss and disease emergence [4]. One socio-ecological phenomenon that has caught a great amount of attention is the dynamics of deforestation [3], and the transition between agricultural and forested land [5], [6], [7]. Transitions in land-use change have been modeled using Markov Chains [5], [6]. Several models have shown that the proportion of forested land can be explained as a function of information flow, adaptive social learning and the rate of deforestation, which can be modulated by the utility of land according to its use, and the forest recovery rate, which is a biological attribute of the landscape [5], [6].

Deforestation has also been long recognized as one of the major drivers for the emergence of infectious diseases affecting humans, with studies documenting the emergence of malaria [8], [9], leishmaniasis [10], [11] and Yellow fever [12] shortly after large scale land use changes. An additional insight from the study of the association between malaria emergence and deforestation was the correlation of malaria endemicity with the formation of latifundia, i.e., the accumulation of land tenure by a small number of landowners, a pattern observed both in the Agro-Pontino Romano for centuries [8], and Spain during the 1930s [13]. More specifically, it has been suggested, and documented, by the long historical records for the Roman Agro-Pontino [8], that deforestation and agricultural development led to ideal conditions for the development of mosquito vectors of malaria parasites [8], a fact biologically instantiated by ecological research over recent years [12]. The debilitating effects of malaria on farmers reduce their ability to harvest crops and lead to the sale or abandonment and adjudication of land by healthier and/or wealthier landowners that will underutilize land as latifundia, i.e., large states whose exploitation, because of the landmass size, require the labor of workers who do not own the land [8]. When land cover is primarily forested, land tenure can be redistributed for agricultural exploitation, and in turn result in a repeated cycle of agricultural exploitation, malaria transmission and latifundia formation [8]. The problem of latifundia formation has widespread consequences, for example, it can be at the basis of biodiversity loss in countries with extreme inequities in wealth, where latifundia and the lack of land property rights are among the major causes behind deforestation [14], [15], [16], [17], [18]. More generally, latifundia are also detrimental to society as demonstrated by the cliodinamical analysis of societies that declined or disappeared after they promoted the creation of latifundia, e.g., ancient Rome [19] or that switched from models of land tenure equity to latifundia, e.g., China at the end of the Tang dynasty [20].

Here, we present a model of latifundia formation that considers the dynamics of land use change, among the following land-use states: forest, agricultural land and land in recovery [5] and the pre-capitalist trade of land in recovery [21]. The pre-capitalist trade implies that land exploitation does not lead to the accumulation of capital, and that goods are traded by their “instantaneous” value [21]. First, we mathematically analyze the case for 2 landowners and then study the case for n>2 landowners through computer intensive simulations. In our model a finite and equal amount of land (which can be in any of the different land use “states”) is divided among a fixed number of landowners which get different utilities from the land they own according to its state. In our model landowners only trade empty land in order to increase their profit. This null-model for latifundia formation successfully recreated patterns of latifundia formation or “land equity”, i.e., a situation where a large proportion of the original landowners remained owning land, once land use and trade reached an equilibrium, i.e., when there were no changes after further model iterations. We then used this model, with parameters that favor “land equity” to test the influence of disease on latifundia formation. We found the assumption of a discounted land utility, i.e., that disease reduce the profits from land use, was a plausible driver of latifundia formation under conditions that would otherwise never lead to inequities in patterns of land ownership.

## Materials and Methods

### Data Patterns

**Figure 1:**
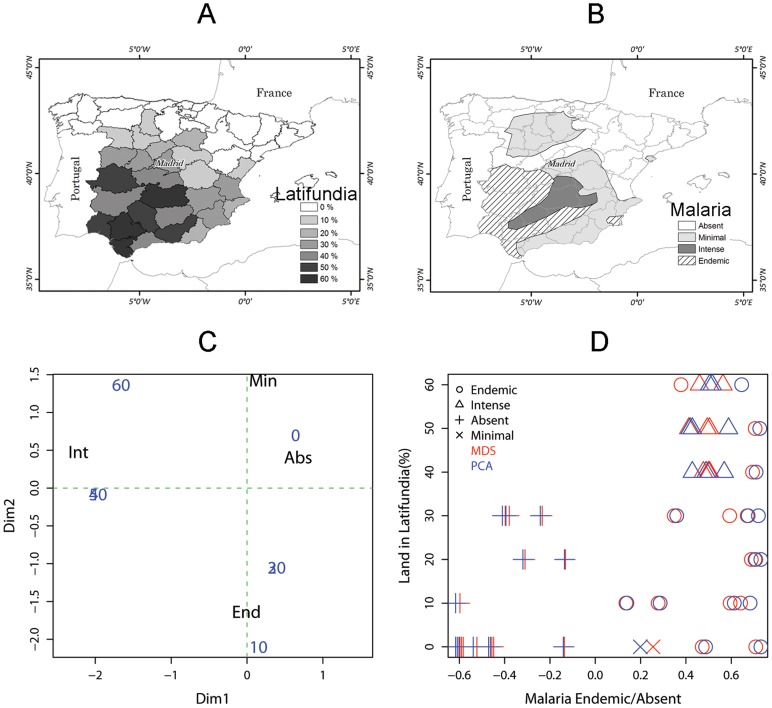
Latifundia, malaria and their association patterns in Spain during the 1930s. (**A**) Percent of land properties that were latifundia, i.e., land properties larger than 25 hectares (**B**) Malaria endemicity (Endemic>Intense>Minimal>Absent) (**C**) Correspondence analysis between malaria endemicity classes (Obtained from a partition around medoids cluster analysis, see Protocol S1 Appendix A for further details) and the percent of latifundia. Malaria categories are (End = endemic, Int = intense, Min = minimal and Abs = absent) (**D**) Percent of Land in Latifundia as function of malaria endemicity indices (the indices were based on the first component of a principal components analysis, PCA or Multidimensional Scaling, MDS, see legend for color and symbol explanation, see also Protocol S1 Appendix B for further details). (A) and (B) are re-drawn from Beauchamp [13].

Figure 1A shows the percent of land exploited as latifundia and Figure 1B the different degrees of malaria endemicity in Spain during the 1930s [13]. Data from the maps in Figure 1A and 1B were extracted using ARCGIS® from the maps in Beauchamp [13]. The association between latifundia, which can also be measured in terms of landmass, such as states larger than 25 ha., i.e., requiring external labor [13], and malaria endemicity, based on a cluster analysis (partition around medioids), for each province was studied using a multiple correspondence analysis between categories for the level of land exploited as latifundia and the dominant malaria endemicity level for each province (see Protocol S1 Appendix A for details). We also derived continuous endemicity indices employing principal components analysis and multidimensional scaling to better visualize the relationship between malaria transmission and land exploited as latifundia (see Protocol S1 Appendix B for details).

Figure 1C shows that epidemic malaria transmission was associated with large proportions of land being exploited as latifundia in Spain during the 1930s. This is supported by the proximity between the categories Int (intense malaria) and values above 40 (which indicate the proportion of land exploited as latifundia). Figure 1D shows how, in general, as malaria transmission intensity increased, the percent of land exploited as latifundia also increased, as well as, its variability. Data patterns indicate that a good null-model of latifundia formation should be able to present a wide variability in the likelihood of latifundia formation in a context with disease transmission (Figure S1).

### Model Details

#### Basic land use model

Let’s assume there is a landscape that is subdivided in **n**×**m** land parcels, where **n** individual landowners possess **m** of the parcels at the beginning of the dynamics. Each land parcel, *k*, belonging to landowner *i*, can transition between three possible states, *S*, at time *t*:
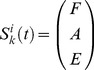
(1)where *F* denotes forested (native vegetation in a wider sense) land, *A* agricultural land and *E* stands for post agricultural land, e.g., land where agriculture is not profitable anymore. The transition from *F* to *A* is governed by a deforestation rate, *r*, from *A* to *E* by a degradation rate, *η*, and from *E* to *A* by a forest recovery rate, *μ* [5]. Assuming changes between land-use types are unidirectional, then the probabilities of staying in the same state is 1−*r* for *F*, 1−*η* for *A* and 1−*μ* for *E*. Although the transition rates between different land uses can be modeled to change as function of global characteristics of the landscape [6] or information flux [7] we will fix these rates to ease the understanding of latifundia formation.

#### Basic Land Trade Model

To study the dynamics of land ownership and the mechanisms of latifundia formation, we can add a layer of complexity to the previous model by incorporating the trade between individual landowners. To incorporate the trade between individual landowners, we define a trade rate (*t_ji_*) as the probability of individual *i* purchasing land from individual *j.* If we further assume that land trade is restricted to parcels in the *E* state, and that trade happens before land transition is realized, we can obtain the following equations for the amount of land belonging to an individual landowner *i* trading with *j*≠*i*:
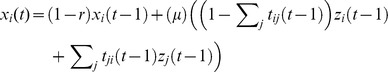


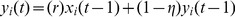
(2)

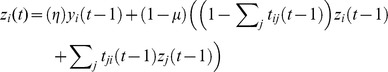



**Figure 2:**
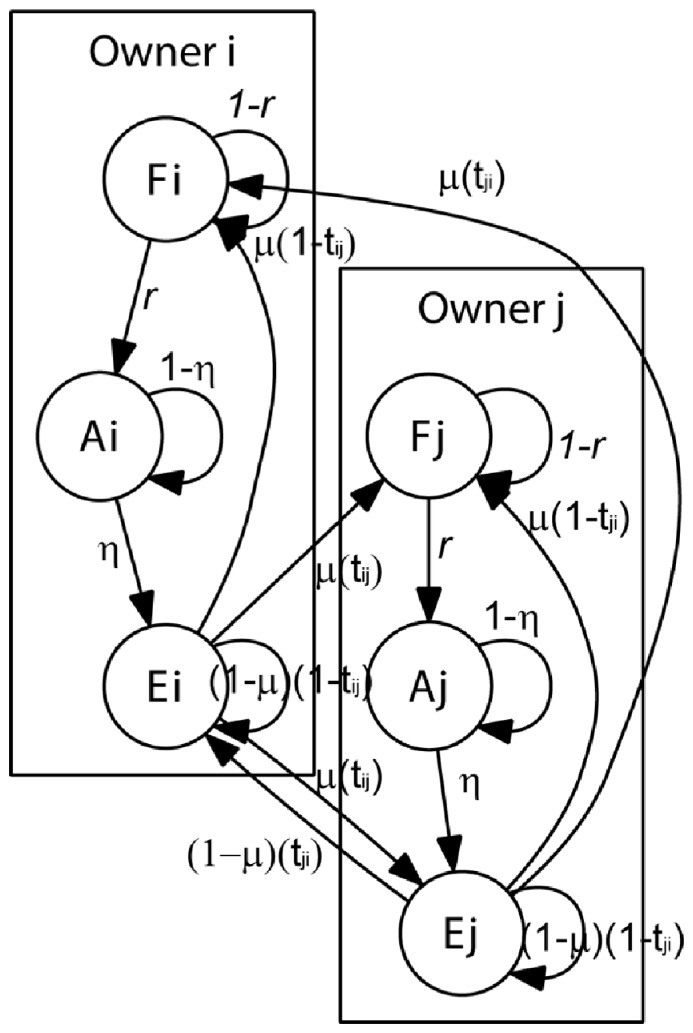
Model description. Graph of land use transition and trade between Owner *i* and Owner *j*, for a detailed explanation of the transitions see equations 1 & 2 in the methods section.

This model is illustrated in Figure 2, which shows how land changes status according to its use, and how land can be traded between owner *i* and *j*. Equation (2) allows the estimation of land in the different states belonging to a given individual *i* at a given time, i.e., *x_i_(t)*, *y_i_(t)* and *z_i_(t)* respectively represent the amount of land that is forested, under agricultural production or in a post-agricultural stage at a given time *t*. *t_ij_* the probability of individual *i* selling land to individual *j.* Finally, *t_ji_*, *μ, η* and *r* parameters already defined in the text preceding equation (2).

#### Defining the trade rates

The trade rates will be a function of the purchasing power (*P_i_*), which is the probability of acquiring land at a given time by a landowner *i* and the sale pressure (*V*_j_) which is the probability of selling land by a landowner *j*≠*i*. Let’s further assume that purchasing ability, *P_i_*, is a function of the utility, *u*, of each parcel, *k*, belonging to each individual, *i,* at time *t*:
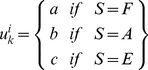
(3)where *a* indicates the forest value attributed to ecosystem services when the parcel is forested, *b* represents the monetary benefits by crop sales minus the cost of agricultural land management; *c* is the utility for a post-agricultural parcel. We define purchasing ability (or power), *P_i_*, as the probability of a landowner to purchase land in relation to other landowners as defined by his/her assets in relation to those present in the whole population:



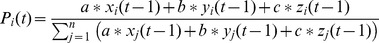
(4)For the sale pressure individuals in a population of landowners can use several criteria. First, we will consider when the sale pressure is the complement of the purchasing power. Under this scenario how likely a landowner is to sell his land is the complement of his/her purchasing power:
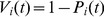
(5)


Under this assumption landowners with a higher purchasing power (*P_i_*) are less likely to sell their land. This assumption is analogous to the one behind the territory size increase in the Colllins-Turchin model of geopolitics [22], where the more powerful (richer) state (landowner) is more likely to conquer (buy) land from the weaker (poorer) state (landowner). Two additional cases that we considered are presented in Protocol S1 Appendix C.

#### Model analysis in the case of two landowners i and j

As a first approach we can study the case of two landowners *i* and *j* presented in Figure 2. For trade to occur one landowner needs to sell land, and another needs to buy it. Let’s assume, the trade from *i* to *j*, *t_ij_* is defined by the purchase ability of *j*, *P_j_*, and the sale pressure of *i* is the complement of the purchase power, i.e., (1−*P_i_*). Furthermore equation (5) implies that *P_i_+P_j_ = *1, therefore:
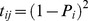
(6)

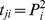
(7)which can be used to find a steady state solution (see Protocol S1 Appendix D) for the system of equations presented in (2):



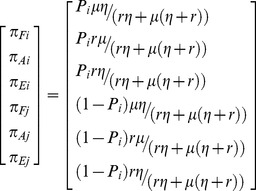
(8)From equation (8) we have that *P_i_*>1−*P_i_* implies that land, independent of its use, will spend more time in the hands of landowner *i*, and if *P_i_* = 1 all land belongs to landowner *i.* Only when *P_i_* = *P_j_* = 0.5 a land equity equilibrium can be expected.

Results of equation (8) assume that *P_i_* and *P_j_* are fixed through time. However, *P_i_* and *P_j_* can change through time because the total utility from the land of a given landowner can change through time following equation (4). When *P_i_* follows the dynamics of equation (4), and *V_i_* follows the dynamics of equation (5), we have that when *P_i_(t = 0)*>*P_j_(t = 0)*:
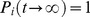
(9)meaning that when a landowner begins with an advantage in his/her utilities when compared with the other landowner he/she will become the latifundist as time goes on. The demonstration can be seen in the Protocol S1 Appendix D. This result also holds when *V_i_* follows the assumptions presented in Protocol S1 Appendix C.

#### Model analysis for more than two owners

**Figure 3:**
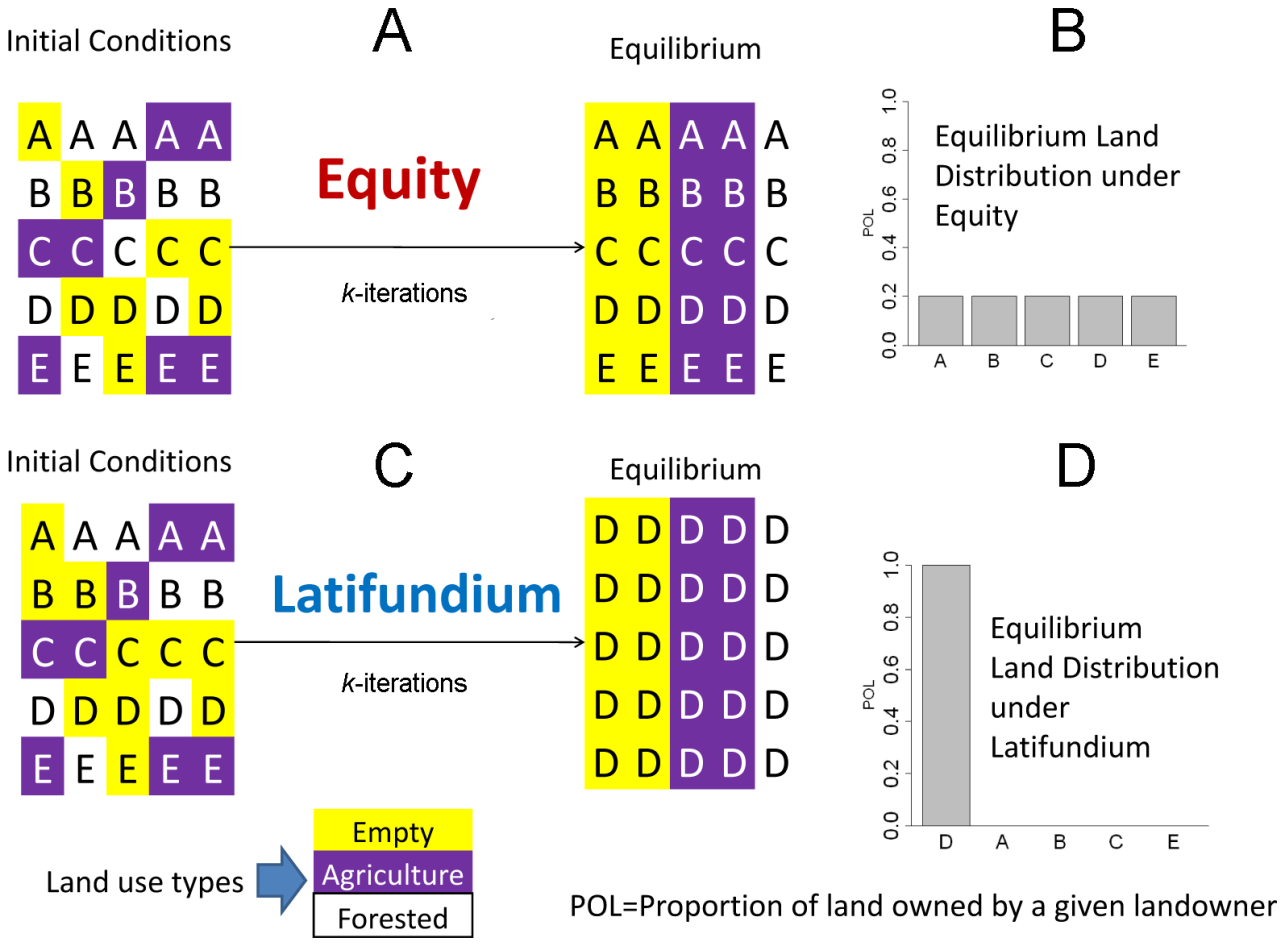
Model simulation scheme. At the beginning of the simulations all landowners have the same amount of land, however the state of the parcels is randomly assigned, after k iterations the model can converge to: (**A**) an equilibrium of equity, where all landowners have the same amount of land which is at equilibrium regarding the land use transitions, which implies (**B**) an uniform distribution in land ownership or (**C**) a Latifudium equilibrium, where all the land (in equilibrium regarding land use transitions) belongs to a single landowner, which implies (**D**) a skewed distribution with one (or a few) landowners accumulating land ownership. In (A) and (C) letters represent different landowners, and colors the land use, see figure legend for further details.



In reality, landscapes tend to be divided among more than two landowners. Although *P_i_(t = 0)*>1/2 is a general condition for latifundia formation that extends to cases with more than two landowners (i.e., **n**>2) we need to resort to computationally intensive simulations to understand the sensitivity of the dynamics to different system sizes (both increasing the number of landowners, **n**, and land parcels per landowner, **m**) and to different transition rates along the different land uses (*S,* see equation 1), as well as differences in the utilities of the different land types (*u*, equation 3). Thus, we performed a series of simulations to understand what conditions were the most likely to promote land equity and latifundia. In all simulations we generated the different initial conditions by assuming that for each individual landowner the amount of land in each state came from two uniform distributions whose mean was equal to 1/3 of the amount of land per capita considered in the system, and the third state was the difference to complete **m**, the number of land parcels per capita. We then iterated the equations presented in (3) until the model reached a steady state (i.e., the values of *x_i_, y_i_* and *z_i_* kept constant through time for the **n** landowners) and then we computed whether the system reached an equilibrium of equity or converged into a latifundia (Figure 3). In the simulations land parcels were assumed to be a continuous variable, i.e., parcel fractions could be traded. To ease model implementation at each time step we computed a pool of the land on sale:

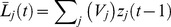
(10)which was then redistributed according to each individual purchasing power (*P_i_*):



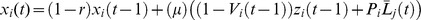


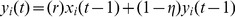
(11)

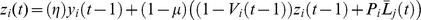



To ease the interpretation of the model, we assumed that each simulation iteration corresponds to one year.

#### Incorporating disease dynamics

To test the hypothesis that disease transmission can promote or enhance latifundia formation [8] we coupled a Susceptible-Infected-Susceptible pathogen transmission model [23] to the land trade model. In this model we assumed that all individuals that were infected at a given time step will recover the next year and then we fixed the basic reproductive number (*R_0_*) of the disease. We assumed disease transmission to be frequency dependent so that:
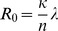
(12)where *κ* was the number of infected individuals the previous year, *n* the total population size and *λ* the force of infection. Thus, depending on the variable number of *κ* we were able to estimate *λ* as follows:



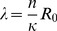
(13)This *λ* was used to assign an infected status to some of the landowners by the simple rejection simulation method, i.e., whenever a random value from a uniform distribution was more extreme than *λ* we identified an individual as infected. When an individual was infected the utilities of his land parcels were discounted by a parameter *h*, which represents the decreased productivity of a sick landowner. *h* defined in (0,1) and equation (4) was rewritten as follows for an infected individual:
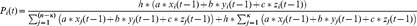
(14)


And:
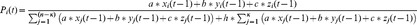
(15)


For an individual free of infection. In (14) and (15) *κ* is the number of infected individuals in the previous time step. In the models dealing with disease we employed equation (5) for the sale pressure.

In the land trade model with discounted utility for disease infected landowners we inquired whether differences in disease susceptibility increased the likelihood of latifundia formation by making some individuals insensitive to infection and comparing the results with simulations were such protection was absent in the population. To further understand the impacts of disease we proceeded with two kind of simulations, in one case we introduced the disease in a population where land equity was an initial condition (a state were no latifundia can be formed) and tested whether disease transmission was able to generate latifundia formation in the system. We also studied the impact of having a given proportion of the population protected from the disease on the dynamics of Latifundia formation. In all simulations we assumed the disease to either be endemically established (*R_0_*>1) or epidemic (*R_0_*≈1).

## Results

**Figure 4:**
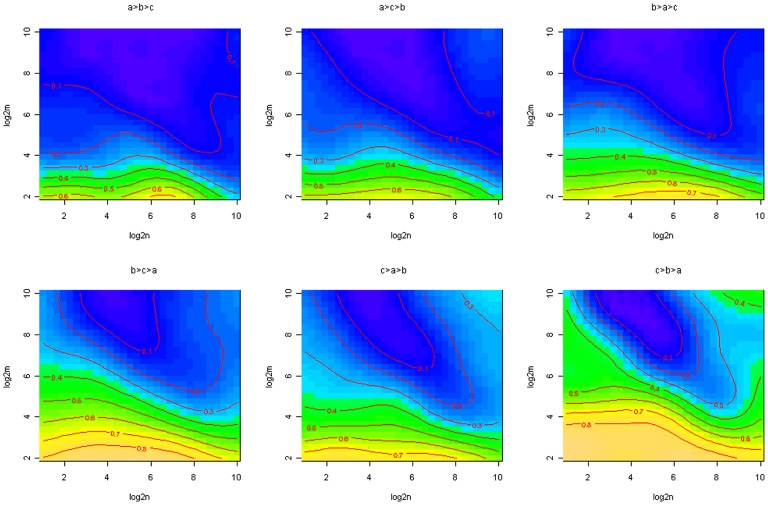
Impact of utility combinations on the likelihood of equity as equilibrium. In top of each panel the relations between the utilities is presented: *a* = forest, *b* = agriculture and *c* = empty (see equation 3 for further details). In each panel, the y axis represents the log_2_ of the number of parcels (**m**) per individual and the x axes present the log_2_ of the number individuals (**n**). Simulations were run 100 times for each combination of **n** and **m**, where the values of n and m were 2 to the power of the values in the x and y axes respectively. Contour lines give the probability of equity as equilibrium for a given parameter combination. Contour lines were obtained with a generalized additive model where the probability of latifundia formation was a smoothed function of the number of parcels and individuals in the model. In all the simulations run to draw this figure the transition rates across land use types were fixed equal to 0.5, i.e, *μ = η = r = *0.5. Regarding the utilities of each land type they were always 3, 2 and 1 for any sequence of *u_k1_>u_k2_>u_k3_*, where 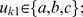 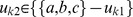 and 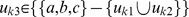. In all panels blue corresponds to low probability of equity and green to high probability of equity.

Simulations from our model (Figure 4) showed that a combination of utilities were empty land had the highest utility (denoted by *c* in equation 3), followed by the utility of agricultural land (*b* in equation 3) and forested land (*a* in equation 3), was the best combination of utilities able to produce a null model were latifundia was less likely to occur. As suggested by the data on malaria transmission and land in latifundia from Spain in the 1930s (Figure 1), the model where the utilities are ranked *c* = 3>*b* = 2>*a* = 1 is a good null model to test the effects of disease transmission on latifundia formation. This ranking of land use utilities can create scenarios where simulations from our model are less likely to generate latifundia.

We found that when sale pressure was defined as the complement of the purchasing power, latifundia were less common in the outputs from our model simulations. Figure S2 and S3 show, respectively, the outcomes for the case when landowners make decision for land sale based on the average or median landowner assets. These last two assumptions were more likely to promote the formation of latifundia under the conditions considered in our simulations.

**Figure 5:**
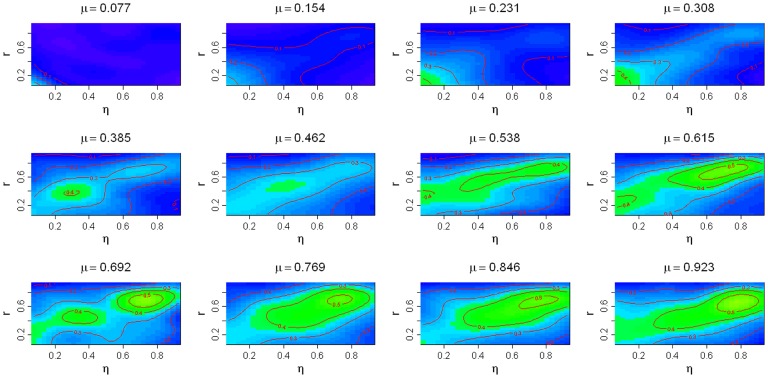
Sensitivity analysis to changes in the transition rates between land use. In the top of each panel the degraded land recovery into forest rate (*μ*) is presented, the x axis represents the agricultural land degradation rate (*η*) and y axis deforestation rate into agricultural land (*r*). Simulations were run 100 times for each combination of *μ, η* and *r.* Contour lines indicate the probability of equity for a given parameter combination. Contour lines were obtained with a generalized additive model where the probability of latifundia formation was a smoothed function of the rates considered in the x and y axis. In all the simulations run to draw this figure the number of parcels per individual (m) and the number of individuals in the population were fixed to 64, a quantity that we assume to reflect the structure of rural communities in ancient Rome [19], [22] (see Figures S4, S5, S6, S7 for results with other values of m and n). Utilities were as follows: *c* = 3, *b* = 2, *a* = 1. In all panels blue corresponds to low probability of equity and green to high probability of equity.

Another important result shown by Figure 4 is that latifundia were more likely to emerge in a larger population of landowners, i.e., the proportion of land exploited as latifundia increased with the number of landowners used for model simulation. A detailed sensitivity analysis of this model showed that low rates of land use change or very different rates of land use change promoted the emergence of latifundia (Figure 5). By contrast, high and similar rates of land use change were associated with the emergence of land tenure equity (Figure 5). In general similar results were observed when the number of parcels or landowners were slightly changed with respect to the values set for Figure 5 (see also, Figures S2, S3, S4, S5). Nevertheless, as population size increased there was an increasing trend in the probability of latifundia formation.

**Figure 6:**
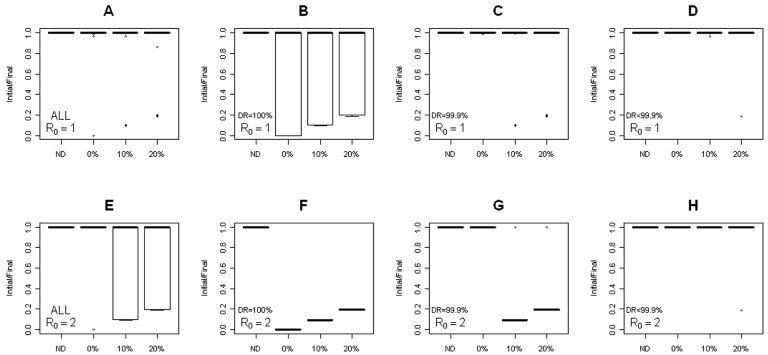
Ratio of initial to final landowners under disease transmission. Panels (A) to (D) show the patterns of latifundia formation (i.e., a low ratio of initial to final landowners) under epidemic conditions (*R_0_* = 1), panels (E) to (H) under endemic conditions (*R_0_* = 2). In Panels (A) and (E) there is no discount rate (DR = 0, h = 1 in equation 13), in panels (B) and (F) there is 100% discount rate (DR = 100, h = 0 in eq. 14), in panels (C) and (G) there a 99.9% DR (h = 0.01 in eq. 14) and panels (D) and (H) show the results for DR<99.9. In all panels the x represents the proportion of landowners that were protected from disease transmission (0, 10, 20) and the control simulation ran under conditions that do not lead to latifundia formation (i.e., perfect equity on land ownership and use, ND in the x axis). In all simulations, parameters for land use change were set up equal to 0.5, number of landowners (n) was 64 and parcels for landowner (m) was also 64. In all the simulations run to draw this figure the transition rates across land use types were fixed equal to 0.5, i.e, *μ = η = r = *0.5. Utilities were as follows: *c* = 3, *b* = 2, *a* = 1.

Figure 6 shows the impacts of disease transmission on the likelihood of latifundia formation. Figure 6A to 6D show the impacts of epidemic disease transmission, i.e., when the basic reproductive number of the disease, R_0_, was close to 1. We observed that extremely high discount rates (very low values for the parameter *h* of equation 13) were able to promote the formation of latifundia (low ratios of initial to final number of landowners). Nevertheless, as the parameter *h* increased (or the discount rate decreased) it was observed that increasing the proportion of people unable to acquire the infection could lead to the formation of latifundia. By contrast, the scenario of endemic disease transmission, i.e., when the basic reproductive number of the disease was higher than 1 (R_0_>>1) showed (Figure 6E to 6H) the extreme condition of *h* = 0 to invariably lead to the formation of latifundia, which was alleviated in the case of heterogeneous populations with individuals protected from transmission. Nevertheless, the final proportion of individual landowners was equal to the proportion of the population protected from disease transmission (Figure 6E to 6H). In both the epidemic and endemic scenarios, a decrease in the utility discount rate for sick landowners resulted in a decreased likelihood of latifundia formation (Figure 6). In synthesis, disease transmission might be a plausible driver for the formation of latifundia, especially when there are high discount rates in land use utility of sick landowners or when there are heterogeneities in susceptibility to infection among the landowners.

## Discussion

Latifundia formation has been a cross cultural phenomenon. From records in the western world, dating back the “Historia Naturalis” of Pliny the Elder who foresaw latifundia as the cause of Roman decline [8], the decline of Tang China in the Oriental world [20], revolutionary movements seeking equity in land ownership in Latin America [24], and post-colonial struggles for land access in South Saharan Africa [25] latifundia formation remains an obstacle to social equity, and is linked with major environmental issues, especially deforestation and its associated loss of species diversity [14], [15], [18]. Therefore, understanding the processes underlying latifundia formation remains fundamental to propose land ownership policies that avoid their generation. Our model of land tenure dynamics successfully reproduced patterns of land ownership observed in Spain in the 1930s [13], where a variability in the degree of latifundia formation can emerge from slightly different initial conditions. The different analyses we performed show that our model is more likely to lead to the formation of latifundia as the number of landowners increases. This pattern is interesting as it could reflect some historical facts. For example, China during the Tang dynasty and its immediate predecessors implemented the *juntien* land system which guaranteed equity in land tenure, since following the death of a landowner only 20% of his/her land will be inherited by his/her descendants, and the remaining 80% will be redistributed in the population according with the productivity of each individual able to work the land [20]. This system and other policies promoting socio-economic equity within classes have been suggested as pillars for the success of Tang dynasty as one of the most advanced societies ever [26]. Nevertheless, this system, that avoided the formation of latifundia, collapsed briefly after the An Lushan rebellion, which occurred at a time of demographic changes that significantly increased the size of the Chinese population [27]. This population expansion could have acted together with the fragmentation of land resulting from the juntien [20]. Thus, although our model did not explicitly consider population growth, our simulations show a likely outcome: latifundia might be more likely to emerge in larger populations. The second important inference from our model is the importance that land use transition speed could have on the formation of the latifundia. High and similar transition rates between different land uses also prevented the formation of latifundia, which is a pattern that could explain the absence of latifundia establishment after the colonization of new agricultural land. For example, in the North American Midwest farms have never reached the size that would make them latifundia [28] and this may be related to the similarity between the native prairie vegetation and the agricultural exploitation of crops like corn and wheat. At any rate, the transitions between different land uses are likely similar in the American Midwest.

Our model does not account for other kind of historical phenomena which have created latifundia, for example, the unequal distribution of land following colonialism [24], [25]. Nevertheless, our model can explain the maintenance of these unequal systems of land tenure. In fact, our model shows that small differences in land utilities can lead to the formation of latifundia even if a set of landowners begin with similar amount of land, and as we showed mathematically for the case of two landowners, when a landowner has an initial advantage, it is expected that he/she will eventually own all the land in the system. Therefore, the practical application of our model results is that land redistribution reforms need not only to ensure equity on the quantity of land a landless farmer receives, but also on the utility associated with the potential land uses.

Our model was also able to show the plausibility of disease as driver of latifundia formation, a hypothesis originally suggested by Angelo Celli in the 1930s [8]. Our simulations showed that two key elements of Celli’s original hypothesis are, indeed, fundamental to the formation of latifundia: (i) the decreased utility of land exploitation by the reduced labor ability of sick landowners (which we modeled with the parameter *h*) and (ii) differences in the risk to acquire infections. In that sense our model can mechanistically confirm one of the main observations made by Celli [8], i.e., that people that were protected from disease transmission were more likely to either conserve and/or purchase land from people that were susceptible to disease transmission. Our model specifically showed that a high discount rate in the presence of disease can lead to formation of latifundia independently of the endemic or epidemic status of a disease, and that heterogeneities in disease susceptibility can further increase the likelihood of latifundia formation even if the discount rates in land utility are not 100% for sick landowners. From an applied perspective these results suggest that inequities in the protection against a disease, for example the use and access to disease prevention devices, can promote further socio-economic inequities in societies where a disease is endemically persistent or has frequent epidemics. This phenomenon has been observed in malaria, where inequities in access to insecticides treated nets can feed positive feedback loops further increasing socio-economic differences within a host population [29], [30].

Finally, in summary, our model was able to show: (i) that latifundia can emerge when there are initial patterns of inequity in land ownership, with landowners with the most resources likely acquiring land from landowners with less resources, (ii) that high transition rates in land use can hamper the formation of latifundia while slow transitions can enhance its formation, (iii) having the highest utility in traded land can also regulate the formation of latifundia and (iv) that both heterogeneities in disease susceptibility and high discount rates on land utility by sick landowners can also promote and enhance the formation of latifundia.

## Supporting Information

Figure S1 **Boxplot for malaria endemicity (index based on the 1^st^ PC of land under different malaria transmission endemicity levels) as function of endemicity categories.**
(PDF)

Figure S2 **Exploring Alternative Rules for the Sale Pressure: Sale pressure based on the average of landowners assets.**
(PDF)

Figure S3 **Exploring Alternative Rules for the Sale Pressure: Sale pressure based on the median of landowners assets.**
(PDF)

Figure S4 **Sensitivity analysis to changes in the transition rates between land use and different** ***m*** **(number of landparcels) and** ***n*** **(number of landowners)** ***n*** **= 64,** ***m*** **= 32.** For interpretation and other parameter values see legend of figure 5 in the main text.
(PDF)

Figure S5 **Sensitivity analysis to changes in the transition rates between land use and different** ***m*** **(number of landparcels) and** ***n*** **(number of landowners)** ***n*** **= 64,** ***m*** **= 128.** For interpretation and other parameter values see legend of figure 5 in the main text.
(PDF)

Figure S6 **Sensitivity analysis to changes in the transition rates between land use and different** ***m*** **(number of landparcels) and** ***n*** **(number of landowners)** ***n*** **= 32,** ***m*** **= 32.** For interpretation and other parameter values see legend of figure 5 in the main text.
(PDF)

Figure S7 **Sensitivity analysis to changes in the transition rates between land use and different** ***m*** **(number of landparcels) and** ***n*** **(number of landowners)** ***n*** **= 128,** ***m*** **= 128.** For interpretation and other parameter values see legend of figure 5 in the main text.
(PDF)

Protocol S1 **Appendices.**
(PDF)
