# Supplementary figures and images for: The Dynamics of Latifundia Formation
Source: PLoS One. 2013 Dec 20;8(12):e82863. doi: 10.1371/journal.pone.0082863 (PMC3869715; doi:10.1371/journal.pone.0082863)

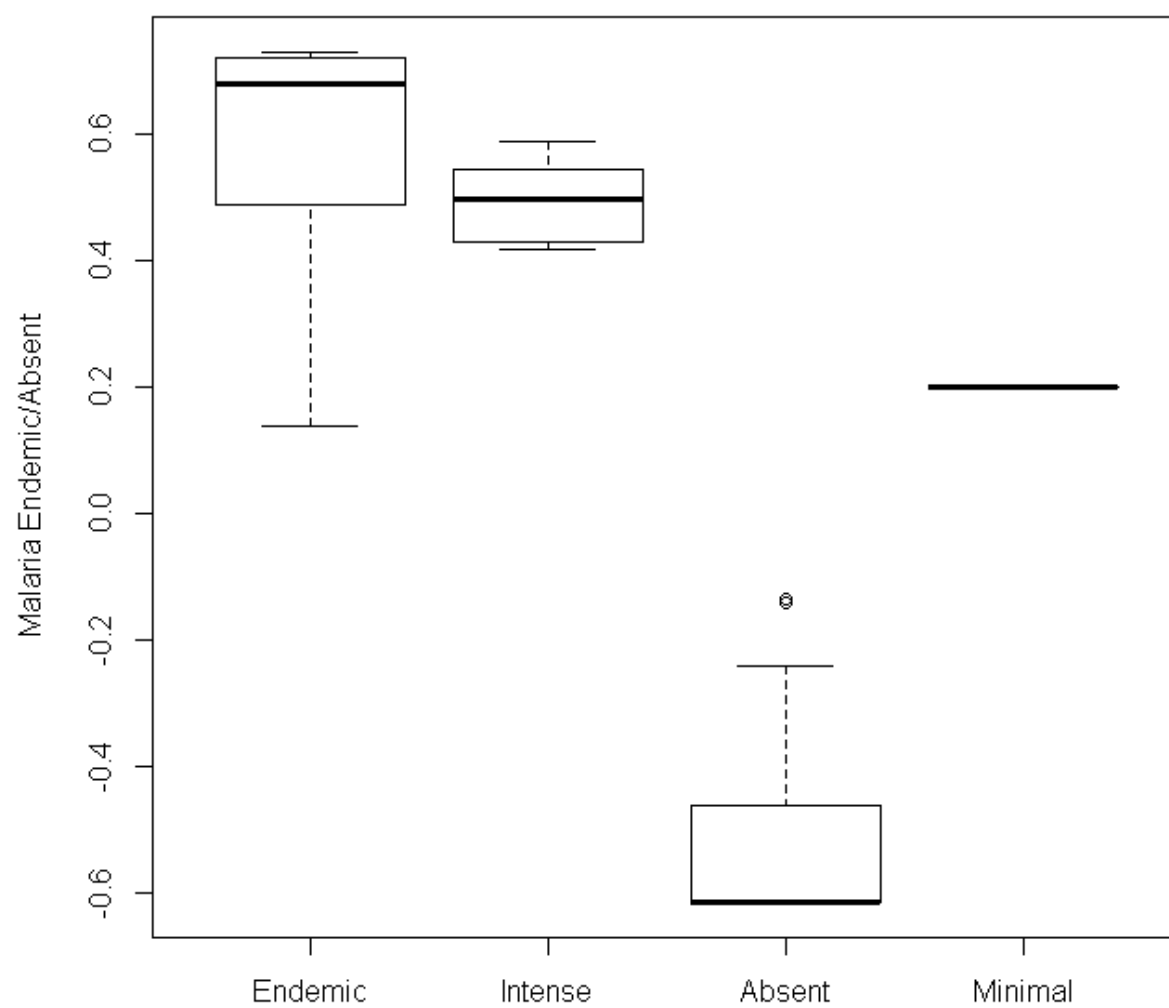

Supplement: Figure S1 — Boxplot for malaria endemicity (index based on the 1st PC of land under different malaria transmission endemicity levels) as function of endemicity categories. (PDF) [file pone.0082863.s001.pdf]

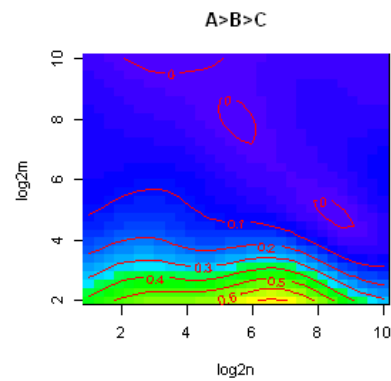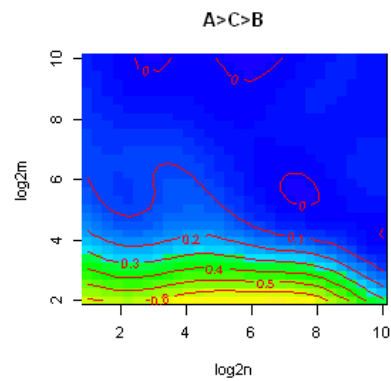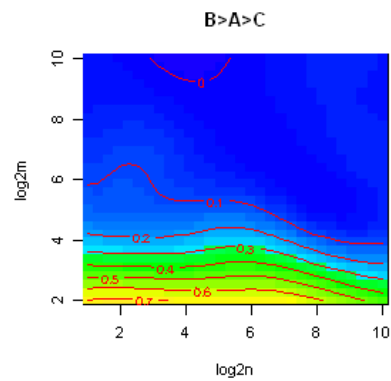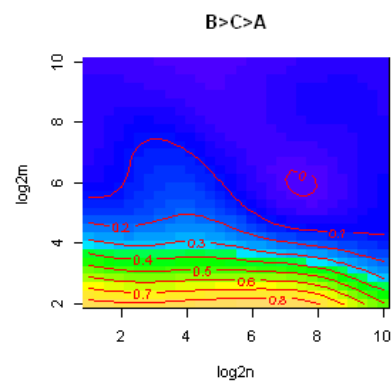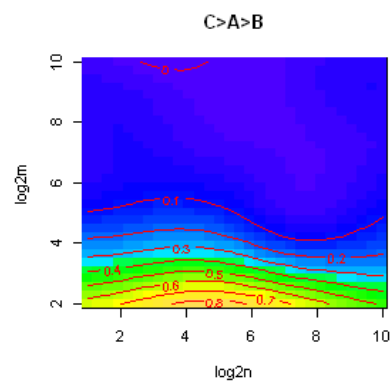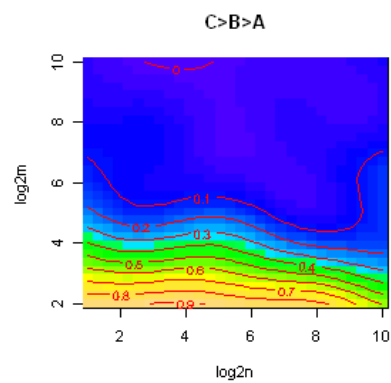

Supplement: Figure S2 — Exploring Alternative Rules for the Sale Pressure: Sale pressure based on the average of landowners assets. (PDF) [file pone.0082863.s002.pdf]

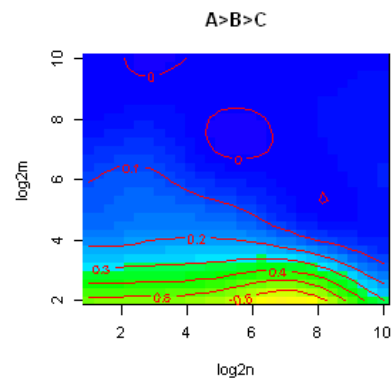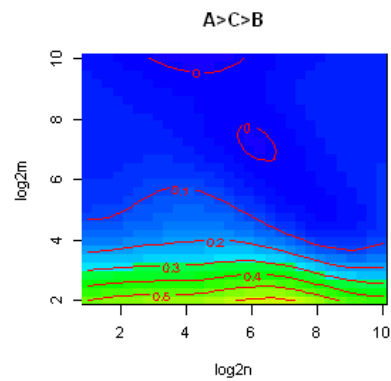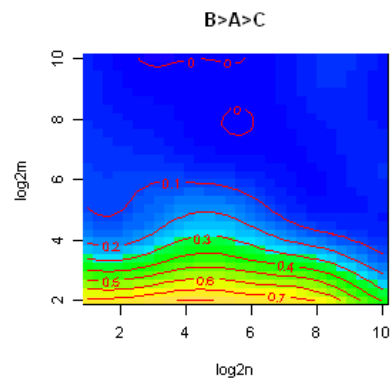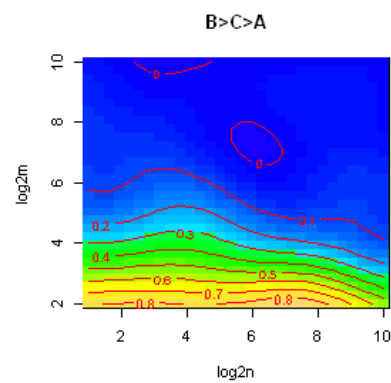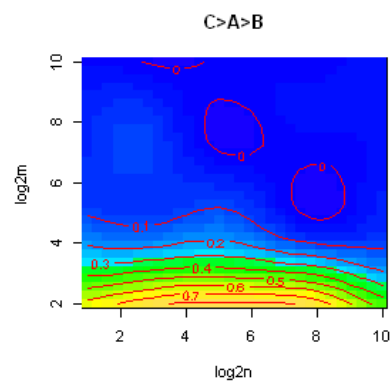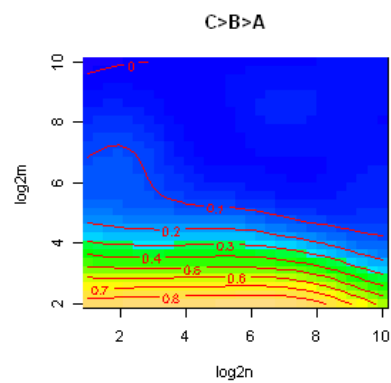

Supplement: Figure S3 — Exploring Alternative Rules for the Sale Pressure: Sale pressure based on the median of landowners assets. (PDF) [file pone.0082863.s003.pdf]

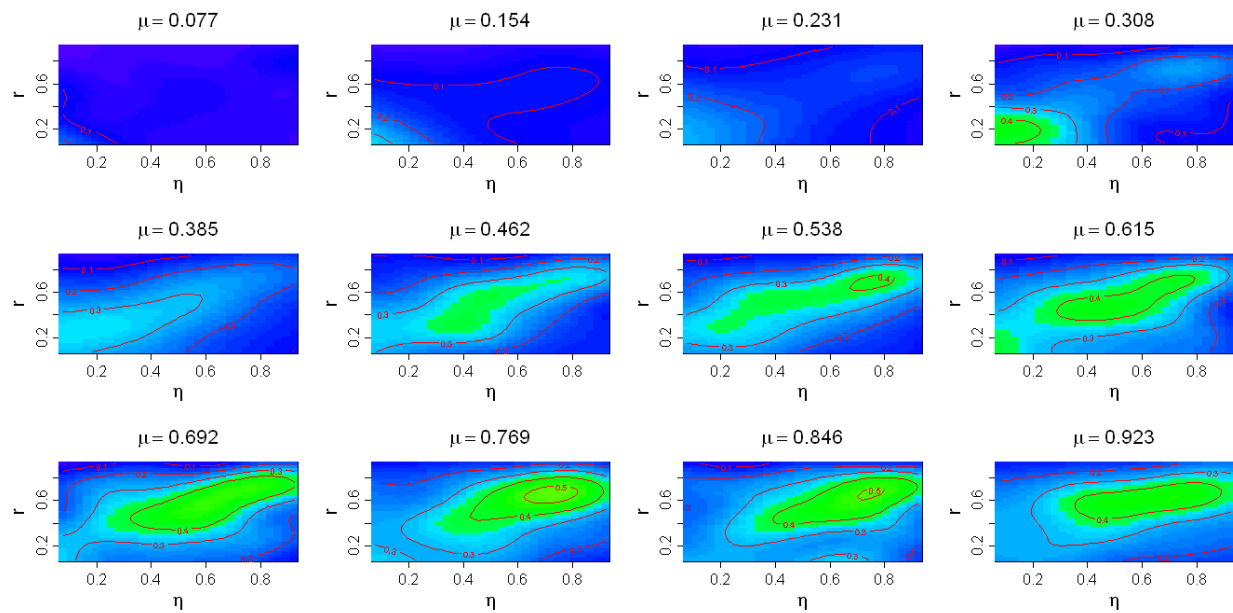

Supplement: Figure S4 — Sensitivity analysis to changes in the transition rates between land use and different m (number of landparcels) and n (number of landowners) n = 64, m = 32. For interpretation and other parameter values see legend of figure 5 in the main text. (PDF) [file pone.0082863.s004.pdf]

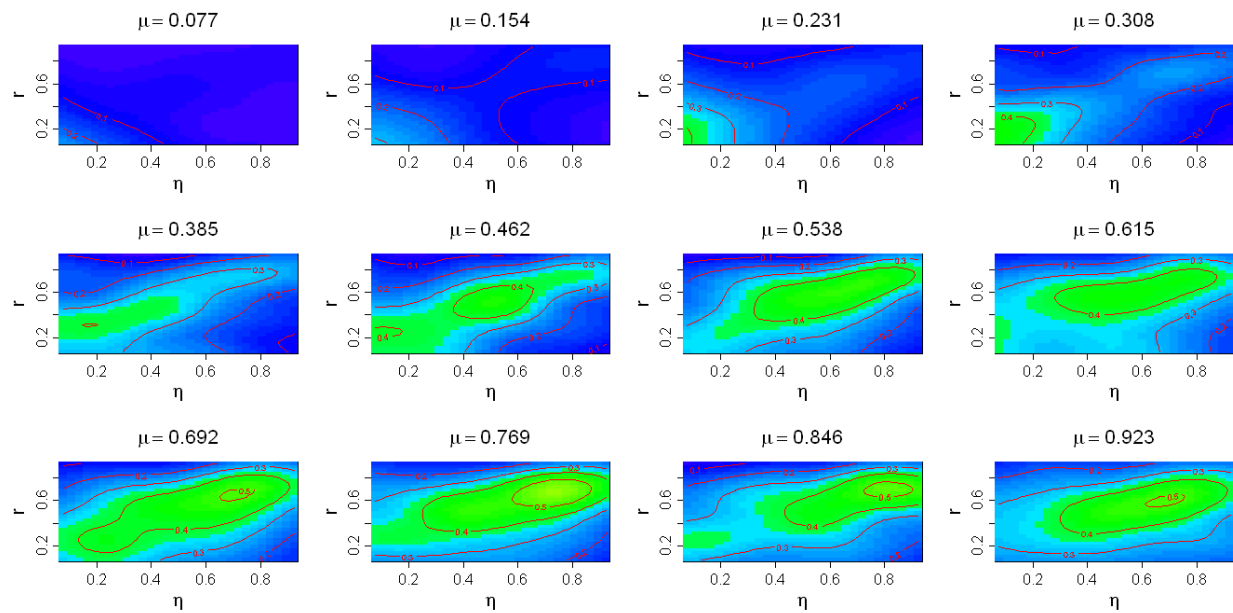

Supplement: Figure S5 — Sensitivity analysis to changes in the transition rates between land use and different m (number of landparcels) and n (number of landowners) n = 64, m = 128. For interpretation and other parameter values see legend of figure 5 in the main text. (PDF) [file pone.0082863.s005.pdf]

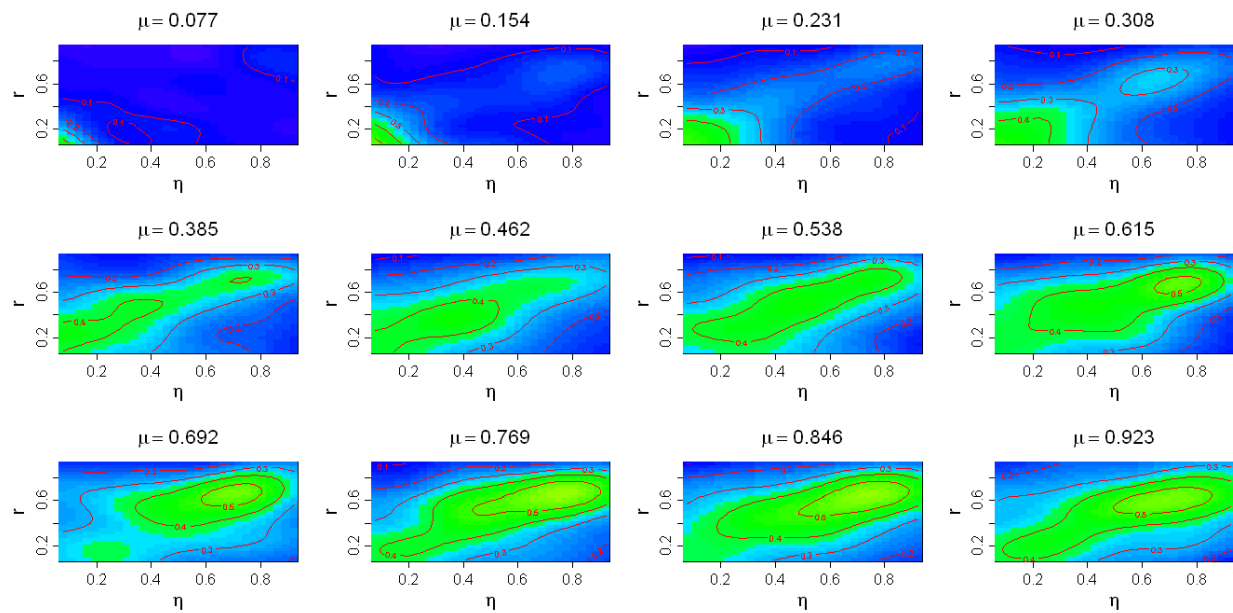

Supplement: Figure S6 — Sensitivity analysis to changes in the transition rates between land use and different m (number of landparcels) and n (number of landowners) n = 32, m = 32. For interpretation and other parameter values see legend of figure 5 in the main text. (PDF) [file pone.0082863.s006.pdf]

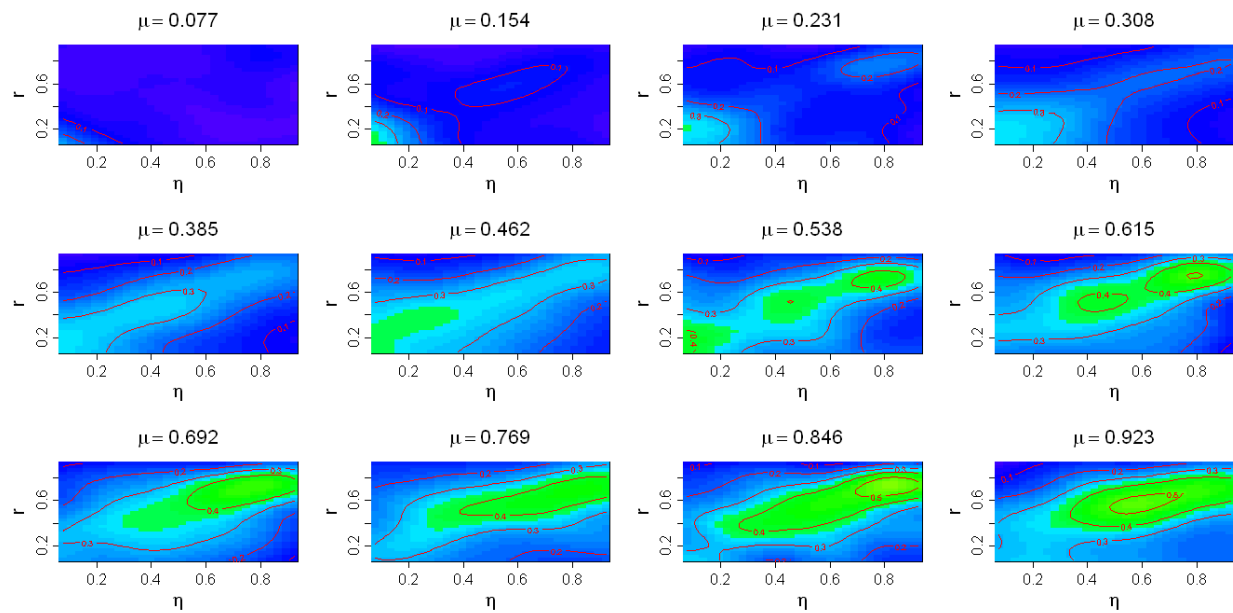

Supplement: Figure S7 — Sensitivity analysis to changes in the transition rates between land use and different m (number of landparcels) and n (number of landowners) n = 128, m = 128. For interpretation and other parameter values see legend of figure 5 in the main text. (PDF) [file pone.0082863.s007.pdf]
