# Supplementary material for: The Dynamics of Latifundia Formation
Source: PLoS One. 2013 Dec 20;8(12):e82863. doi: 10.1371/journal.pone.0082863 (PMC3869715; doi:10.1371/journal.pone.0082863)
Supplement: Protocol S1 — Appendices. (PDF) [file pone.0082863.s008.pdf]

Protocol S1 Appendices

A) Partition around medoids (PAM) cluster analysis for assigning an endemicity class to each province and correspondence analysis (CA) between malaria endemicity and latifundia in Spain during the 1930s.

The original maps of Beauchamp (1985) had data about % of land in latifundia and malaria transmission intensity (or endemicity) presented in different ways. The % of land in latifundia was presented for each province, making unnecessary any elaborated process for data extraction at the province level. However, the endemicity was presented as continuous across provinces borders, thus requiring the use of tools for image analysis to estimate the endemicity level for each province. To assign a malaria endemicity class to each province of Spain we estimated the percent of land covered by each of the four categories of endemicity (Endemic>Intense>Minimal>Absent) in each province using ArcGis. The extracted data was then analyzed using a PAM cluster analysis. In this analysis a number, *k*, of preset clusters is assigned a priori and then clusters are constructed around *k* representive points of the observations in the dataset (Struyf et al, 1997). The number of clusters selected by cross-validation for PAM was four, which were able to separate 4 levels of endemicity that roughly corresponded to the four categories presented in Figure 1B (See also Appendix B). We then performed a CA to study the association between the level of malaria endemicity and land in latifundia of each province. Briefly, a CA is a multivariate analysis that can measure the association between categories of different variables (Brand, Numerical Ecology), then when variables are projected in a plane spatial proximity is a measurement of association, i.e., the closer two categories are in the projection plane, the more associated they are.

B) Principal component analysis (PCA) and multidimensional scaling (MDS) to estimate an index of malaria transmission based on the malaria endemicity categories presented by Beauchamp (1985) for Spain in the 1930s.

To obtain a continuous measurement of endemicity at each province we performed a PCA and a MDS on the matrix containing the % of land under each Malaria transmission endemicity level. PCA is, among other things, a dimension reduction technique where a data matrix undergoes an orthogonal linear transformation resulting in new coordinates for each object in the original matrix, where the first axis, a.k.a., component, capture the highest amount of variability in the data. To estimate the PCA we employed the variance/covariance matrix of the data. For the PCA analysis we first performed an analysis on the four categories of malaria endemicity, which lead to null loadings for the "intense" category a signal of an inadequate analysis. Thus, we performed a new analysis joining the "ïntense" and "minimum" category. In this second analysis the first component explained 68% of the variability in the data and loadings were as follows: % absent = -0.815, % endemic = 0.229 and % minimum and Intense = 0.532. These loadings imply that more positive values in the first principal component are associated with increased malaria transmission and negative values with the absence of the disease. We performed the MDS,

another dimension reduction technique, to verify that the PCA was an appropriate tool for dimension reduction, since results of PCA and MDS should be very similar if relationships between the data are strictly linear (as observed in Figure 1D). For the MDS we employed data from the four categories of malaria endemicity and reduced the data to one dimension. Figure S1, shows a boxplot of the PCA scores as function of the endemicity levels obtained with PAM (see Appendix A). The Figure shows that the PCA based endemicity index is good to separate the four transmission categories (Endemic, Intense, Minimal, Absent).

C) Other rules for the Sale Pressure

The Sale Pressure (V) depends on the relative utilities of landowner i with respect to the average or median utilities of the population.

Under this scenario how likely a landowner is to sell all his land in *E* stage depends on whether his/her utilities are above or below the average:

$$V_i(t)=\begin{cases} 1 & if \ \frac{n*\left(a*x_i(t-1)+b*y_i(t-1)+c*z_i(t-1)\right)}{\sum_{j=1}^{n} a*x_j(t-1)+b*y_j(t-1)+c*z_j(t-1)} < 1 \\ 0 & if \ \frac{n*\left(a*x_i(t-1)+b*y_i(t-1)+c*z_i(t-1)\right)}{\sum_{j=1}^{n} a*x_j(t-1)+b*y_j(t-1)+c*z_j(t-1)} > 1 \end{cases} \qquad \text{(C.1)}$$

Or the median of assets in the population:

$$V_i(t)=\begin{cases} 1 & if \ \frac{\left(a*x_i(t-1)+b*y_i(t-1)+c*z_i(t-1)\right)}{\text{Median Utility}} < 1 \\ 0 & if \ \frac{\left(a*x_i(t-1)+b*y_i(t-1)+c*z_i(t-1)\right)}{\text{Median Utility}} > 1 \end{cases} \qquad \text{(C.2)}$$

D) Finding the stationary state of the land trade model of equation (2)

The probability transition matrix of the Markov chain presented in (2) can be written as follows:

$$\mathbf{P} = \begin{bmatrix} (1-r) & r & 0 & 0 & 0 & 0 \\ \eta & (1-\eta) & 0 & 0 & 0 & 0 \\ \mu P_i & 0 & (1-\mu)P_i & \mu(1-P_i) & 0 & (1-\mu)(1-P_i) \\ 0 & 0 & 0 & (1-r) & r & 0 \\ 0 & 0 & 0 & \eta & (1-\eta) & 0 \\ \mu P_i & 0 & (1-\mu)(P_i) & \mu(1-P_i) & 0 & (1-\mu)(1-P_i) \end{bmatrix} \begin{matrix} F_i \\ A_i \\ E_i \\ F_j \\ A_j \\ E_j \end{matrix} \qquad \text{(D.1)}$$

By the Erdos-Fellerd-Pollard Theorem, since the Markov chain is irreducible, aperiodic and recurrent we can compute the proportional time spend in each state ($\prod$)

$$\begin{bmatrix}\pi_{Fi}\\ \pi_{Ai}\\ \pi_{Ei}\\ \pi_{Fj}\\ \pi_{Aj}\\ \pi_{Ej}\end{bmatrix} = \begin{bmatrix}(1-r) & 0 & \mu P_i & 0 & 0 & \mu P_i \\ r & (1-\eta) & 0 & 0 & 0 & 0 \\ 0 & \eta & (1-\mu)P_i & 0 & 0 & (1-\mu)(P_i) \\ 0 & 0 & \mu(1-P_i) & (1-r) & 0 & \mu(1-P_i) \\ 0 & 0 & 0 & r & (1-\eta) & 0 \\ 0 & 0 & (1-\mu)(1-P_i) & 0 & \eta & (1-\mu)(1-P_i)\end{bmatrix} \begin{bmatrix}\pi_{Fi}\\ \pi_{Ai}\\ \pi_{Ei}\\ \pi_{Fj}\\ \pi_{Aj}\\ \pi_{Ej}\end{bmatrix} \quad \text{(D.2)}$$

Which provided:

$$1 = \pi_{Fi} + \pi_{Ai} + \pi_{Ei} + \pi_{Fj} + \pi_{Aj} + \pi_{Ej} \quad \text{(D.3)}$$

Can be solved as shown in equation (7)

E) Demonstration that $P_i(t=0)>P_j(t=0)$ implies $P_i(t\rightarrow\infty)=1$

When two landowners share the land in a landscape $P_i(t)+ P_j(t)=1$, therefore $P_j(t) =1 - P_i(t)$. $P_i(t)>P_i(t-1)$ if $P_i(t)=P_i(t-1)+\varepsilon$. The latter is true when ε is positive, which requires:

$$\mathbf{z_i(t)-z_i(t-1)>0} \quad \text{(E.1)}$$

Substituting the trade rates of equation (6) in equation (2), we have that (E.1) is true when:

$$[(1-\mu)P_i(2-P_i)-1]z_i(t) + (1-\mu)(P_i)^2 z_j(t) > 0 \quad \text{(E.2)}$$

Which is always true for any value of z_j provided that $\mu < 1$ in the Markov chain definition. (E.2) is true for z_i when:

$$P_i(2-P_i) > \frac{1}{1-\mu}$$

$$(P_i)^2 - 2P_i + \frac{1}{1-\mu} < 0$$

$$P_i(t) < 1 \pm \sqrt{\frac{\mu}{1-\mu}}\, i \quad \text{(E.3)}$$

Which is always true for the real part, because $P_i \leq 1$ by definition. The bounded growth, in a probability space, for condition $P_i(t)>P_i(t-1)$ implies that:

$$P_i(t\rightarrow\infty)=1 \quad \text{(E.4)}$$
